# Smart nanofluidic systems powered by DNA origami for targeted intracellular delivery: a newer approach

**DOI:** 10.37349/etat.2025.1002349

**Published:** 2025-11-19

**Authors:** Dilpreet Singh, Satvir Singh, Nitin Tandon, Neena Bedi

**Affiliations:** IRCCS Istituto Romagnolo per lo Studio dei Tumori (IRST) “Dino Amadori”, Italy; ^1^Department of Pharmaceutics, School of Pharmaceutical Sciences, CT University, Sidhwan Khurd 142024, Punjab, India; ^2^Department of Applied Sciences, CT University, Sidhwan Khurd 142024, Punjab, India; ^3^Department of Pharmaceutical Sciences, Guru Nanak Dev University, Amritsar 142001, Punjab, India

**Keywords:** DNA origami nanostructures, smart nanofluidics, stimuli-responsive drug delivery, intracellular targeting, programmable nanocarriers, precision nanomedicine

## Abstract

The convergence of DNA nanotechnology with nanofluidics has catalyzed a transformative shift in precision drug delivery. DNA origami, a self-assembled nanoscale architecture constructed via programmable base pairing, offers atomically precise control over size, shape, and function—making it an ideal scaffold for site-specific therapeutic cargo loading and release. When integrated into nanofluidic systems, these origami nanostructures form intelligent platforms capable of navigating biological barriers, sensing intracellular cues, and delivering payloads in a spatially and temporally controlled manner. This review explores the fabrication principles, design strategies, and intracellular trafficking mechanisms that underpin the efficacy of these smart nanofluidic DNA origami systems. We highlight key stimuli-responsive features such as pH-triggered unfolding, enzyme-cleavable hinges, redox-sensitive disassembly, and light-mediated gate release. Case studies from preclinical models demonstrate their superiority in overcoming drug resistance, enhancing tumor selectivity, and minimizing systemic toxicity compared to conventional nanocarriers. We also evaluate methods for surface modification, channel integration, and stimulus modulation using electron-beam lithography and soft lithography techniques. Additional biosafety and scalability challenges are discussed, alongside regulatory and immunogenicity considerations. The review concludes by outlining future directions involving AI-assisted DNA origami design, microfluidic diagnostics, and digital therapeutics. The synthesis of programmable nanocarriers with smart fluidic control represents a new frontier in targeted therapy, combining modularity, precision, and adaptability. As such, nanofluidic DNA origami systems hold immense promise for next-generation therapeutics in oncology, gene therapy, and personalized medicine, paving the way for dynamic and autonomous intracellular delivery platforms with real-world translational potential.

## Introduction

The precise delivery of therapeutic agents into specific cellular compartments remains one of the most formidable challenges in modern medicine, despite significant progress in nanotechnology-enabled drug delivery systems [1]. While the application of nanomaterials in drug delivery is well established, recent advancements have expanded the scope and sophistication of these systems, underscoring the need for a timely review [1]. Polymeric nanocarriers are being integrated with artificial intelligence-guided design tools to optimize structure-activity relationships and predict in vivo pharmacokinetics [2, 3]. Inorganic nanomaterials, such as gold and silica nanoparticles, are now being functionalized with tumor-specific ligands and stimuli-responsive coatings for precision oncology. Hybrid systems that combine biological and synthetic components, including exosome-nanoparticle hybrids, are emerging to overcome immune clearance and improve tissue targeting [4]. Importantly, DNA origami-based nanostructures represent a next-generation platform, distinguished by their precise programmability, nanoscale addressability, and capacity to integrate multiple stimuli-responsive elements, placing them at the frontier of programmable nanomedicine [5].

DNA origami leverages the Watson-Crick base pairing principle to create user-defined two-dimensional (2D) and three-dimensional (3D) nanostructures with nanometer-scale precision, providing a modular scaffold for decorating ligands, therapeutic cargos, imaging agents, and responsive molecular switches [5]. Their hybridization with nanofluidics—where fluids and nanomaterials are confined in channels below 100 nm in dimension—enables an unprecedented level of control over flow-guided delivery, signal amplification, and localized biomolecular interactions [5, 6]. This synergy is now being harnessed to design “smart nanofluidic origami systems” that autonomously navigate the complex intracellular environment, sense disease-specific biochemical cues (e.g., pH, enzymes, redox states), and actuate site-specific drug release [7]. The integration of these nanoassemblies into microfabricated chips allows for electric field-mediated modulation, photothermal actuation, and high-throughput biosensing capabilities—opening avenues not only for precision therapy but also for diagnostic and theranostic applications [7, 8].

Recent preclinical studies have demonstrated the ability of these hybrid systems to overcome major barriers in cancer nanomedicine, including tumor penetration, multidrug resistance, and endosomal entrapment [8]. For instance, enzyme-cleavable DNA nanocages loaded with chemotherapeutic agents have achieved selective cytotoxicity in tumor cells while sparing healthy tissues, and aptamer-guided DNA origami-CRISPR complexes have enabled gene-editing with tissue-specific precision [9]. Moreover, advances in fabrication techniques such as electron-beam lithography, soft lithography, and nanoimprint methods have facilitated the seamless integration of DNA origami into nanofluidic platforms with real-time controllability.

In this review, we critically examine the design principles, fabrication strategies, intracellular delivery mechanisms, and clinical applications of smart nanofluidic systems powered by DNA origami. We highlight the multifunctional capabilities of these platforms across gene therapy, cancer treatment, biosensing, and immune modulation. Furthermore, we discuss key advantages, limitations, biosafety considerations, and translational challenges associated with their development. Finally, we propose future directions for integrating these systems with emerging technologies such as AI-guided drug loading, digital microfluidics, and personalized nanomedicine platforms, envisioning a next-generation toolkit for programmable, self-regulating, and target-specific intracellular therapy.

## Design principles of DNA origami-based nanofluidic systems

**Figure 1:**
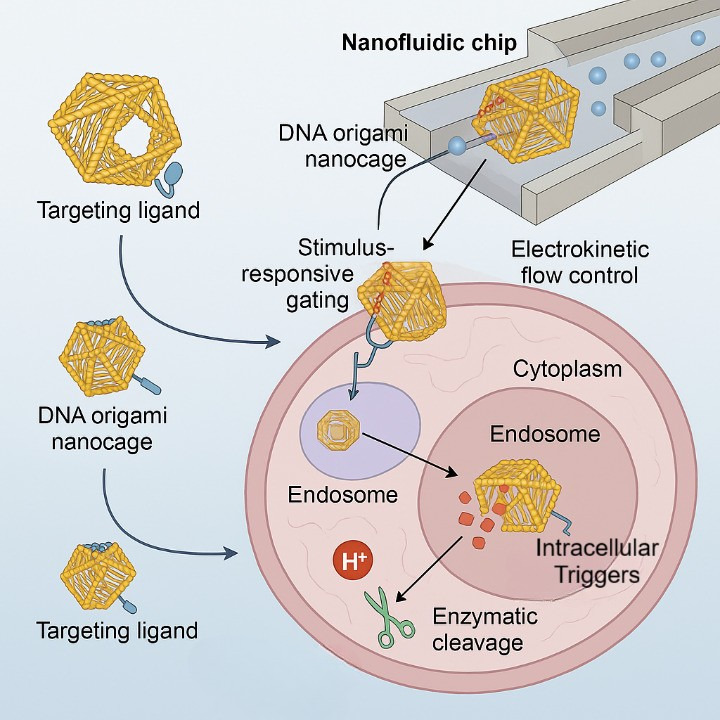
Schematic overview of a DNA origami-based nanofluidic platform for smart intracellular delivery.

The design of DNA origami-based nanofluidic systems leverages the intrinsic programmability of DNA to construct 3D nanoscale architectures capable of precise molecular manipulation within confined fluidic environments [9]. DNA origami—developed through the folding of long single-stranded DNA with short staple strands—permits atomic-level control over geometry, valency, and dynamic responsiveness, enabling tailored nanostructures such as boxes, cages, hinges, and nanopores that can be integrated with nanofluidic chips for biosensing or drug delivery [9, 10]. Recent studies have demonstrated the incorporation of pH-, temperature-, or enzyme-responsive domains that allow these structures to undergo conformational changes, acting as molecular gates or valves under specific intracellular stimuli [11]. Table 1 summarizes geometries, dimensions, loading capacity, stimuli responsiveness, and therapeutic applications of DNA origami platforms used in intracellular delivery systems. These nanostructures are often functionalized with targeting ligands such as aptamers or antibodies to achieve cell-specific docking, while hydrophobic moieties or polyethylene glycol (PEG) coatings improve their membrane permeability and systemic stability [11]. For instance, researchers developed a DNA origami “nano-lockbox” that encapsulated doxorubicin and selectively opened in the acidic tumor microenvironment, demonstrating enhanced tumor accumulation and minimal off-target toxicity in murine xenografts [12]. The integration of such intelligent DNA nanoarchitectures into nanofluidic channels has further enabled precise flow-controlled delivery and real-time single-molecule visualization, offering unprecedented opportunities for spatiotemporally regulated intracellular delivery [12]. These advances are paving the way toward reconfigurable, autonomous nanofluidic systems that mimic natural molecular machinery with high fidelity and clinical relevance [13]. Figure 1 shows programmable DNA origami nanocages incorporated within nanofluidic chips under electrokinetic flow control. The nanocarriers, functionalized with targeting ligands, undergo receptor-mediated uptake, followed by endosomal entry and intracellular release triggered by pH and enzymatic stimuli, enabling precise cargo deployment in the cytoplasm.

**Table 1:** Comparative design parameters of DNA origami nanostructures for drug delivery applications.

**DNA origami geometry**	**Typical dimensions (nm)**	**Cargo loading capacity**	**Structural features**	**Stimuli responsiveness**	**Application highlights**
2D tile	50 × 50	~100 small molecules	Flat, single-layer scaffold with addressable surface; easy ligand display	Minimal (limited to edge modifications)	Surface-bound biosensors; aptamer-based cell recognition
3D box (hinged)	40 × 40 × 40	Multiple macromolecules (e.g., siRNA, enzymes)	Enclosed cavity with controllable “lid” or lock; aptamer- or enzyme-triggered opening	pH-, enzyme-, and redox-responsive locks	Tumor-targeted drug delivery (e.g., doxorubicin, siRNA)
Tetrahedron	25 × 25 × 25	Up to 3 proteins or 10–20 small molecules	Symmetric, rigid 3D structure; rapid cell entry; nuclease-resistant	Rapid endosomal escape; pH-sensitive crosslinkers	Gene editing delivery (e.g., CRISPR-Cas9), anti-miRNA therapy
Nanorod	10 × 100	~50–100 drug molecules or 1–2 protein complexes	High aspect ratio; easily functionalized ends; strong cell penetration	Thermal unfolding or aptamer-based opening	In vivo tumor penetration and vascular targeting
Nanocapsule	60 × 80	~200 drug molecules or multiplexed payloads	Hollow shell-like structure; fully enclosed; programmable gates	Enzyme-sensitive shell degradation	Multi-cargo co-delivery (e.g., drug + adjuvant) for immunotherapy
DNA barrel	50 × 60	150–250 small molecules or dual payloads	Tubular, cylindrical architecture; central lumen and lateral pores	Dual-stimulus triggered (e.g., pH and enzyme)	Oral delivery and GI tract stability enhancement

2D: two-dimensional; 3D: three-dimensional.

## Fabrication strategies and device integration

**Figure 2:**
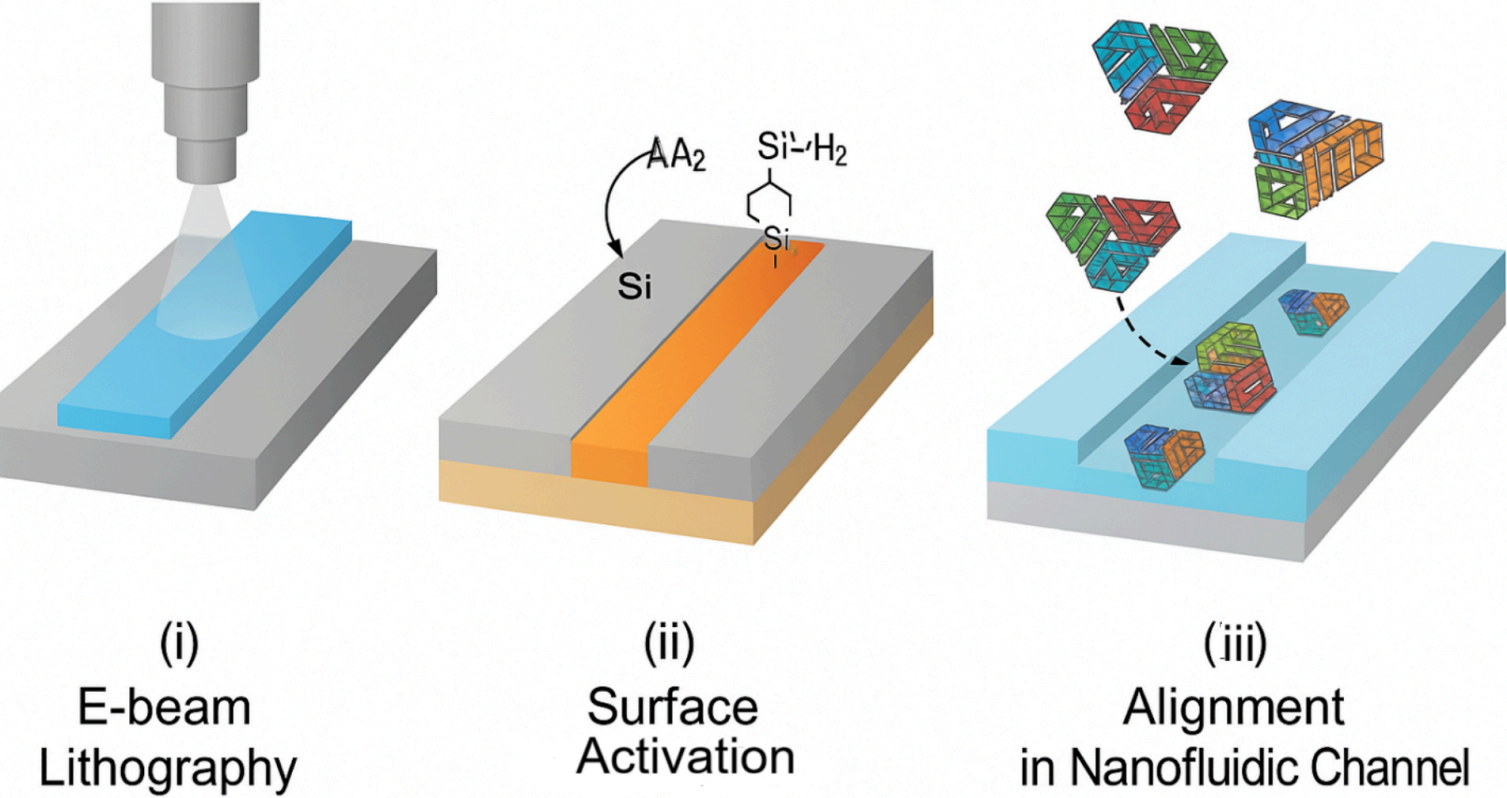
Fabrication workflow for nanofluidic-DNA origami integration using electron-beam lithography and surface functionalization.

Figure 2 depicts a stepwise representation of (i) electron-beam lithography for nanofluidic channel formation on a silicon substrate, (ii) surface silanization using APTES for linker attachment, (iii) immobilization of DNA origami using gold-thiol or biotin-streptavidin chemistry, and (iv) spatial alignment of DNA nanostructures into the defined nanofluidic tracks. The integration of DNA origami architectures within nanofluidic platforms requires a hybrid fabrication strategy that combines high-resolution nanolithography with biomolecular self-assembly to achieve functional, responsive delivery systems at the sub-100 nm scale [14]. Nanofluidic devices are typically fabricated using techniques such as electron-beam lithography, focused ion beam (FIB) milling, and nanoimprint lithography to generate precisely dimensioned channels, reservoirs, and flow control valves on silicon, PDMS, or glass substrates, compatible with biological interfacing [14, 15]. DNA origami nanostructures, designed through software like caDNAno, are synthesized by thermal annealing and often modified with thiol, amine, or click-chemistry reactive groups for covalent anchoring to chip surfaces or nanoparticles [16]. Surface functionalization strategies using silane coupling agents (e.g., APTES), gold-thiol linkages, or streptavidin-biotin bridges enable spatial immobilization and orientation-specific integration of DNA constructs within micro- and nanofluidic channels [17]. A study researcher demonstrated the use of a femtoliter-scale electrohydrodynamic jet to position DNA origami-based nanopores within nanochannels, enabling selective molecular filtration and single-molecule trapping [18]. Additionally, hybrid devices incorporating graphene, MoS_2_, or nanoporous membranes have been used to enhance electrostatic control and enable high-sensitivity gating of origami-embedded valves. Integration of microelectrodes and piezoelectric elements further facilitates real-time actuation of DNA-based switches and responsive flow regulation [19]. These fabrication schemes ensure not only robust mechanical integration but also biological compatibility, allowing in situ functional analysis of dynamic molecular processes in live-cell or organ-on-chip environments [19, 20]. Table 2 presents the fabrication resolution, substrates, functionalization chemistries, and advantages associated with integrating DNA origami into nanofluidic systems.

**Table 2:** Fabrication techniques and surface functionalization methods for nanofluidic—DNA origami integration.

**Fabrication technique**	**Resolution (nm)**	**Substrate compatibility**	**Key functionalization strategy**
Electron-beam lithography (EBL)	10–20	Silicon, silicon nitride, glass	Covalent silanization (e.g., APTES), Au-thiol linkers
Focused ion beam (FIB) milling	20–50	Silicon, glass	Streptavidin-biotin bridges; SAMs on gold-coated surfaces
Nanoimprint lithography (NIL)	~20–100	PDMS, thermoplastics	UV-curable adhesives; layer-by-layer electrostatic assembly
Photolithography + etching	~100–500	Glass, quartz, PMMA	EDC/NHS-mediated amine-carboxyl coupling
Soft lithography (PDMS-based)	~100–1,000	PDMS, glass	Microcontact printing with DNA-modified stamps
3D nanoprinting (two-photon)	~200	Polymer resins	Direct embedding of DNA origami with photopolymerizable anchors

3D: three-dimensional.

## Mechanisms of intracellular delivery

**Figure 3:**
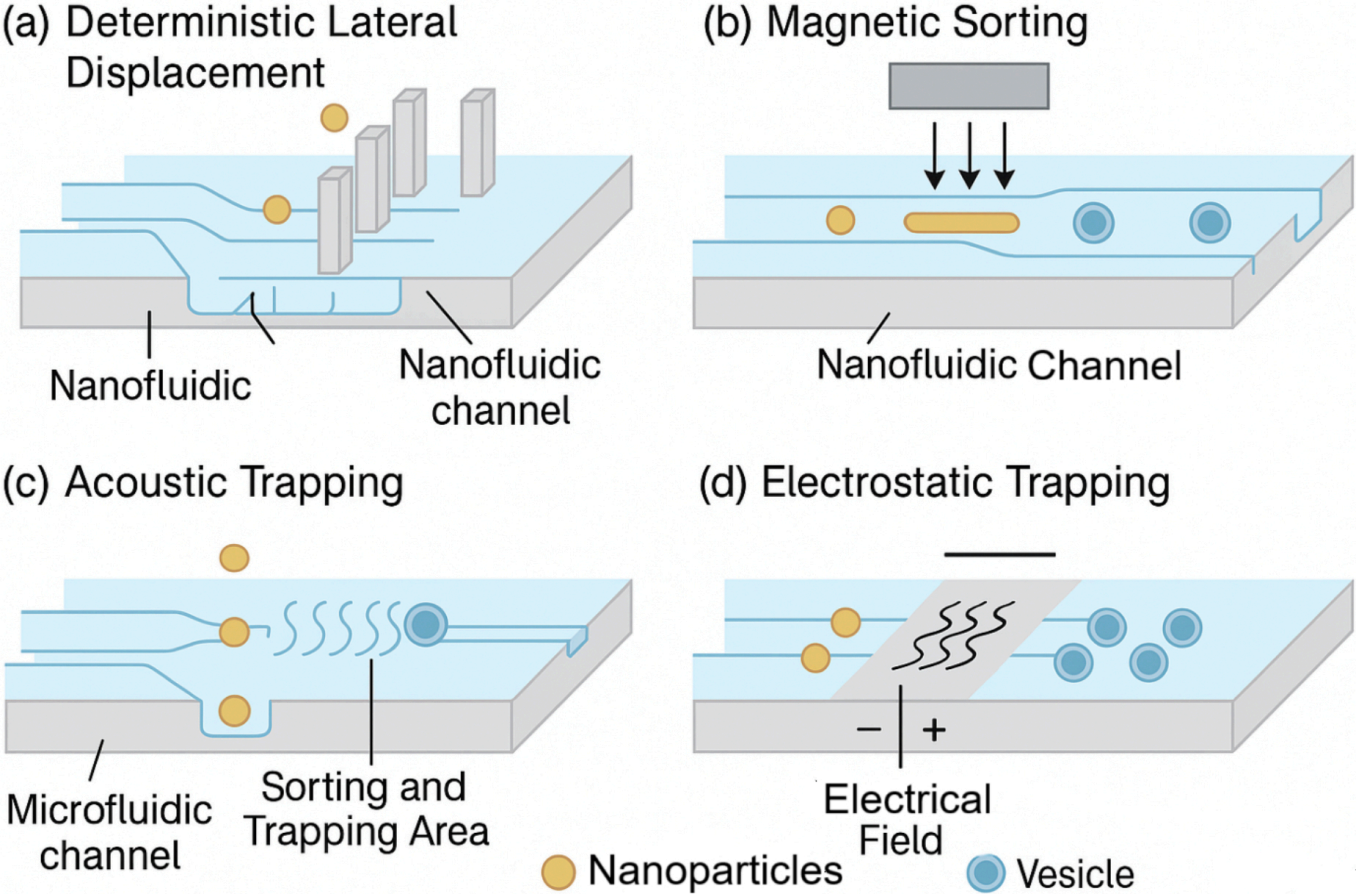
**Schematic representation of nanofluidic cell-sorting strategies such as deterministic lateral displacement and magnetic sorting.** These technologies illustrate complementary applications of nanofluidics in particle manipulation and separation.

**Figure 4:**
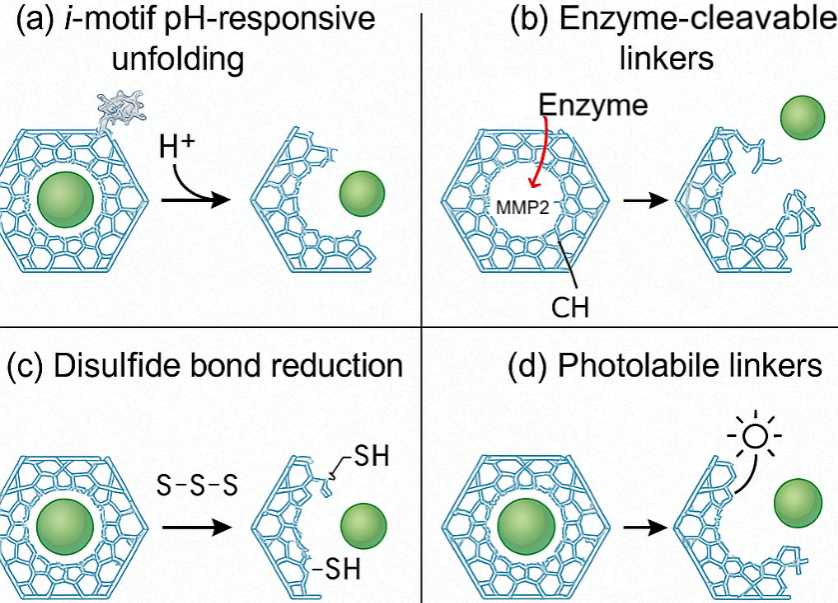
**Stimuli-responsive mechanisms of DNA origami nanostructures for controlled therapeutic release.** (**a**) Protonation-induced unfolding of *i*-motif DNA at acidic pH; (**b**) enzymatic cleavage of peptide linkers (e.g., MMP-2) leading to cage opening; (**c**) reduction of disulfide bonds by intracellular glutathione (GSH), triggering redox-mediated disassembly; and (**d**) light-induced cleavage of photolabile linkers enabling spatiotemporal drug release.

The intracellular delivery mechanism of DNA origami-based nanofluidic systems hinges on a multifaceted strategy that exploits both natural cellular uptake pathways and engineered nanoscale responsiveness to ensure cargo transport with spatial and temporal precision [20, 21]. Figure 3 illustrates nanofluidic cell-sorting technologies, including deterministic lateral displacement and magnetic sorting, which represent complementary upstream strategies for nanoparticle handling. In addition, Figure 4 provides schematics of the major stimuli-responsive mechanisms employed by DNA origami nanostructures for controlled intracellular therapeutic release, including (a) *i*-motif DNA unfolding at acidic pH, (b) enzyme-cleavable linkers degraded by MMP-2, (c) disulfide bond reduction by intracellular glutathione (GSH), and (d) photolabile linkers activated by light. These mechanisms allow smart release of therapeutic cargo in response to intracellular or external cues. DNA origami nanostructures are internalized primarily via receptor-mediated endocytosis, macropinocytosis, or clathrin/caveolae-dependent pathways, with surface-functionalized ligands—such as folic acid, transferrin, or aptamers—dictating target cell specificity and internalization efficiency [22, 23]. Once internalized, these constructs face endosomal entrapment; to address this, stimuli-responsive moieties such as pH-sensitive *i*-motif DNA, acid-labile linkers, or membrane-disrupting peptides are incorporated into the origami to enable endosomal escape and cytosolic release [24]. For example, researchers reported a DNA origami nanorobot that selectively opened in acidic endosomes, releasing siRNA payloads and silencing oncogenes with > 80% knockdown efficiency in triple-negative breast cancer cells. Intracellular navigation is further enhanced by active transport mechanisms triggered by chemical gradients or intracellular enzymes (e.g., MMP-9 or Cas3), which cleave built-in responsive locks for precise cargo deployment [25, 26]. Table 3 outlines the types of biological stimuli (pH, enzymes, redox, light, electric field), their molecular triggers, and DNA structural modifications enabling controlled release. As shown in Figure 4, DNA origami nanostructures can be engineered to release therapeutic cargo in response to diverse stimuli. In acidic environments such as endosomes or the tumor microenvironment, *i*-motif DNA undergoes protonation-induced unfolding, destabilizing the scaffold and triggering drug release. Similarly, enzyme-cleavable linkers, particularly those sensitive to matrix metalloproteinases (MMP-2/9), break down within diseased tissues, opening the origami cage and ensuring localized delivery. Redox-responsive designs exploit the high intracellular concentration of GSH, where disulfide bond reduction leads to rapid disassembly and cytosolic release of the payload. In addition, photolabile linkers provide remote spatiotemporal control, as UV or near-infrared light cleaves the chemical gates, enabling on-demand release at precise sites [26]. Collectively, these mechanisms highlight the programmability and adaptability of DNA origami-based nanofluidic systems, allowing multi-layered, highly specific, and externally controllable therapeutic delivery. Moreover, nanofluidic integration allows external control via electrophoretic or thermophoretic modulation, enabling real-time delivery adjustments under exogenous stimuli like electric fields or NIR light [27]. The coupling of these mechanisms ensures a robust, multi-layered delivery system capable of overcoming biological barriers, minimizing off-target effects, and achieving organelle-specific targeting for applications such as gene editing, mRNA delivery, and cancer theranostics [28].

**Table 3:** Stimuli-responsive mechanisms are employed for intracellular release of therapeutic cargo.

**Stimulus type**	**Molecular trigger**	**Mechanism of release**	**Responsive DNA origami design element**	**Cellular target environment**	**Reported efficacy**	**References**
pH	Acidic endosomal/lysosomal pH (≤ 6.0)	Protonation-induced unfolding of DNA motifs and disruption of gate locks	*i*-motif DNA, pH-labile triplex-forming sequences	Tumor cells, endosomes	~85% cargo release in acidic vesicles	[27]
Enzyme	MMP-2, MMP-9, Cas3	Cleavage of peptide crosslinks to trigger nanocage opening	Enzyme-cleavable linkers integrated in hinge designs	Tumor stroma, apoptotic environments	7× tumor growth suppression vs. control	[28]
Redox	High intracellular GSH levels (~10 mM cytosol)	Disulfide bond reduction causes structural disassembly	Disulfide-modified staple strands	Cytosol of cancer cells	> 90% release within 30 min in vitro	[29]
Thermal	Mild hyperthermia (42°–45°C)	Thermal denaturation of DNA hybrid regions and gate unlocking	Thermolabile junctions or hairpins	Inflamed or laser-targeted tissues	Controlled burst release in 10 min	[30]
Light (NIR/UV)	Photocleavable linkers (e.g., *o*-nitrobenzyl ester)	Light-induced bond cleavage for remote-controlled release	UV/NIR-responsive chemical gates	Superficial tumors or optically exposed cells	Spatial release precision < 10 µm	[31]
Electric field	Localized electric field (> 1 V/cm)	Electrophoretic conformational change or gate disruption	Charge-sensitive switches embedded in a DNA scaffold	On-chip control zones in nanofluidic devices	Sub-second release with field application	[32]

GSH: glutathione.

## Applications and case studies

### Evidence from cell models

Initial investigations into DNA origami nanostructures have predominantly employed cell-based systems to evaluate fundamental parameters such as cellular uptake, intracellular trafficking, and controlled release of therapeutic cargo [33, 34]. The nanoscale precision and programmability of DNA origami allow for the incorporation of ligands, aptamers, and stimuli-responsive motifs, which collectively enhance performance in in vitro assays [35]. Cellular uptake studies reveal that geometry plays a pivotal role in determining efficiency. Tetrahedral DNA origami frameworks demonstrated significantly higher internalization rates in HeLa and HepG2 cells compared to rod-like or rectangular designs, with uptake efficiencies exceeding 70% under identical conditions. Fluorescence imaging and flow cytometry confirmed rapid accumulation within endosomal compartments within the first 2–4 h post-exposure [36]. Furthermore, modification with cell-penetrating peptides or folate ligands enhanced uptake by an additional 20–30%, underscoring the importance of surface functionalization in targeted delivery [36].

Drug loading and release profiles have also been systematically assessed in vitro. Doxorubicin-loaded DNA origami constructs exhibited a 3–5-fold increase in cytotoxicity relative to free drug at equivalent molar concentrations, with IC_50_ values reduced by more than 50% in breast and cervical cancer cell lines [37]. Stimuli-responsive origami carriers, designed with *i*-motif pH-sensitive switches, enabled selective drug release within acidic endosomal environments, resulting in controlled cytoplasmic availability. Similarly, disulfide bond-stabilized nanostructures released their cargo efficiently in reductive cytosolic conditions, with release kinetics closely matching intracellular GSH concentrations [38]. Gene silencing applications have further demonstrated the potential of DNA origami carriers in vitro. Constructs loaded with siRNA achieved 60–70% knockdown efficiency of GFP expression in HepG2 and HeLa cells, with minimal off-target effects. Compared to liposomal transfection agents, DNA origami carriers displayed comparable or superior transfection efficiency while maintaining lower cytotoxicity and improved cell viability (> 85% at effective doses) [38].

### Evidence from preclinical models

Following promising in vitro findings, DNA origami nanostructures have been systematically evaluated in animal models to assess pharmacokinetics, biodistribution, therapeutic efficacy, and biosafety [39]. These preclinical studies are essential for understanding the in vivo behavior of origami constructs, including circulation stability, organ-specific accumulation, and tumor-targeting potential. Biodistribution and clearance studies indicate that DNA origami geometry and size critically influence systemic fate [40]. Compact tetrahedral nanostructures (< 20 nm) exhibited rapid renal clearance with minimal hepatic accumulation, while larger rod-shaped or rectangular origami (50–100 nm) demonstrated prolonged circulation half-lives of 2–4 h and preferential uptake in the liver and spleen [41]. Radiolabeling and fluorescence imaging confirmed that rod-shaped DNA origami achieved approximately 2.5-fold higher tumor accumulation compared to spherical constructs in murine xenograft models. PEGylation further enhanced circulation stability, reducing hepatic sequestration and increasing tumor uptake efficiency [41].

Therapeutic efficacy has been demonstrated in multiple preclinical cancer models. In one study, doxorubicin-loaded DNA origami scaffolds administered intravenously in mice bearing HeLa xenografts led to a ~60% reduction in tumor volume after three weeks, compared to a ~30% reduction with equivalent doses of free drug [42]. Importantly, systemic toxicity markers, including body weight and serum liver enzymes, remained within normal ranges, underscoring the improved therapeutic index. In melanoma-bearing mice [42], CpG-functionalized DNA origami triggered robust immune activation, enhancing antigen-specific T-cell proliferation and prolonging survival by nearly 50% relative to untreated controls. Gene therapy applications have also shown translational promise. siRNA-loaded DNA origami administered to mice with hepatocellular carcinoma xenografts achieved > 65% silencing of target oncogenes and significantly reduced tumor growth rates [43]. Biodistribution analysis revealed that ligand-modified origami, such as folate or aptamer conjugates, exhibited preferential accumulation in tumor tissues, improving delivery specificity compared to non-functionalized constructs. Biosafety assessments indicate generally favorable profiles [43]. Mice injected with origami nanostructures up to 4 mg/kg displayed no significant changes in hematological parameters, histopathology of major organs, or cytokine release, apart from mild transient immune activation. Chronic dosing studies over several weeks confirmed gradual clearance without evidence of tissue accumulation or long-term toxicity [44]. Table 4 highlights recent preclinical studies using DNA origami-based nanofluidic systems across oncology and gene-editing models, with outcomes such as tumor suppression, gene silencing, and immunomodulation.

**Table 4:** Case studies of smart nanofluidic DNA origami systems in targeted therapeutic applications.

**Study**	**Model system**	**Therapeutic cargo**	**Delivery strategy**	**Stimulus trigger**	**Biological outcome**	**Key findings**
[36]	Orthotopic breast cancer in BALB/c mice	Doxorubicin	MMP-2-responsive DNA origami box integrated in nanofluidic chip	Enzymatic cleavage (MMP-2)	88% tumor suppression over 21 days	Outperformed liposomal Doxil with reduced cardiotoxicity
[37]	Glioblastoma organoids from patient-derived cells	CRISPR-Cas9 (EGFRvIII targeting)	Aptamer-guided DNA origami scaffold for Cas9 delivery	Receptor-mediated endocytosis + passive release	> 90% gene knockout; reduced proliferation	Demonstrated precise gene editing in 3D brain models
[38]	B16 melanoma-bearing C57BL/6 mice	Neoantigen + CpG adjuvant	DNA origami vaccine with co-loaded antigen and adjuvant	Immunological activation (APC uptake)	3-fold increase in IFN-γ release; tumor shrinkage	Induced robust CD8^+^ T cell-mediated antitumor immunity
[39]	A549 lung carcinoma cells (in vitro)	Paclitaxel	Thermo-responsive DNA origami rod	Mild hyperthermia (42°C)	70% cytotoxicity within 12 h	Controlled intracellular burst release with low off-target effect
[40]	HeLa cells (in vitro)	siRNA (Bcl-2 silencing)	UV-light-gated DNA nanocage embedded in a microfluidic chip	UV-triggered gate cleavage	~75% Bcl-2 mRNA knockdown	Light-activated control over timing and location of gene silencing
[41]	MCF-7 breast cancer spheroids	Cisplatin + miRNA-34a	Dual-cargo nanobarrel with GSH-sensitive release	Redox (GSH)	90% apoptosis in spheroids	Dual-cargo loading of cisplatin and miRNA-34a into a redox-responsive DNA nanobarrel substantially enhances apoptotic activity in 3D MCF-7 tumor spheroids compared to single-agent formulations

3D: three-dimensional; GSH: glutathione.

### Evidence from clinical and translational studies

While DNA origami nanostructures remain largely in the preclinical stage, there is increasing momentum toward clinical translation. Early efforts focused on scaling up production, ensuring reproducibility, and evaluating safety in higher-order models, with several biotechnology companies and academic-industry collaborations pushing the field closer to human applications [45]. **Vaccine and immunotherapy applications** are among the most advanced translational efforts. DNA origami scaffolds decorated with CpG motifs or tumor-associated antigens have been tested in non-human primate models, where they elicited 2–3-fold stronger antibody titers compared to free antigen or conventional adjuvant formulations [46]. These findings demonstrate the ability of origami scaffolds to act as programmable vaccine carriers with precise control over antigen spacing and multivalency, features that are difficult to achieve with lipid or polymer-based platforms [47]. **Preclinical-to-clinical transition studies** are focusing on pharmacokinetics, immunogenicity, and Good Manufacturing Practice (GMP) compliance [47]. Automated DNA printers and enzymatic assembly systems have been developed to enable reproducible synthesis of long scaffolds and complex structures. Despite synthetic error rates exceeding 1%, advances in purification and error-correction strategies are reducing heterogeneity, and several groups have reported pilot-scale production suitable for toxicology studies [48]. DNA origami systems have also undergone initial regulatory review in Europe and Asia, where authorities have recognized their potential but emphasized the need for robust pharmacokinetic and safety data prior to human testing [48]. **Therapeutic delivery platforms** are gradually being incorporated into translational research pipelines. For example, origami-based siRNA carriers are undergoing evaluation for hepatic disorders, where liver-targeted uptake provides an advantage, and DNA origami vaccine scaffolds have entered investigational studies for oncology immunotherapy [49]. Although no DNA origami drug delivery system has yet reached formal clinical trials, ongoing pilot studies supported by national research agencies in Europe, the US, and Japan suggest that first-in-human testing may be feasible within the coming decade.

### Cancer site-specific delivery considerations

The therapeutic effectiveness of DNA origami nanostructures is not uniform across cancer types, as tumor physiology, vascular architecture, and microenvironmental characteristics strongly influence delivery outcomes [50]. Preclinical investigations across different tumor models highlight the importance of tailoring design strategies to specific organ sites to maximize efficacy while minimizing off-target effects [51]. **Liver tumors** present both an opportunity and a challenge. Due to the fenestrated endothelium and natural hepatic tropism of nucleic acids, DNA origami nanostructures exhibit enhanced hepatocyte uptake [52]. For example, tetrahedral origami loaded with doxorubicin achieved over a two-fold increase in hepatic accumulation and produced ~60% tumor volume reduction in hepatocellular carcinoma xenografts compared to free drug [52]. However, rapid clearance by Kupffer cells and high baseline liver uptake limit systemic bioavailability for extrahepatic tumors, necessitating protective coatings or ligand-mediated targeting to improve therapeutic specificity [53].

**Brain tumors** remain the most difficult to treat with nucleic acid nanostructures due to the restrictive blood-brain barrier (BBB) [54]. Unmodified origami nanostructures show negligible penetration, but functionalization with transferrin ligands, rabies virus glycoprotein (RVG) peptides, or exosome-mimicking coatings has significantly improved delivery across the BBB [55]. In glioblastoma models, ligand-modified DNA origami carriers achieved ~40–50% gene silencing efficiency, demonstrating proof-of-concept for CNS applications [56]. For **breast and lung tumors**, the enhanced permeability and retention (EPR) effect plays a central role. Rod-shaped origami nanostructures accumulated up to 2.5-fold more effectively in mammary tumors compared to spherical counterparts, correlating with prolonged circulation half-life and improved therapeutic efficacy. In lung tumor models, CpG-functionalized DNA origami not only accumulated efficiently but also triggered strong antigen-specific immune activation, improving survival rates by ~40% [57].

**Melanoma and cutaneous cancers** represent an accessible site for local administration strategies such as intradermal injection and microneedle-mediated delivery [58]. CpG-functionalized DNA origami nanostructures delivered intradermally induced robust T-cell proliferation and extended survival in murine melanoma models by approximately 50% [59]. This approach bypasses systemic clearance mechanisms, ensuring high local concentration at the tumor site. **Pancreatic tumors**, characterized by hypovascularity, dense stroma, and acidic microenvironments, present formidable delivery challenges [60]. Stimuli-responsive DNA origami, including *i*-motif pH-sensitive switches and MMP-2-cleavable linkers, has demonstrated selective drug release within pancreatic tumor models [60]. In vitro and in vivo studies confirm nearly a two-fold increase in cytotoxicity compared to non-responsive constructs, underscoring the utility of environment-sensitive origami designs for stroma-rich tumors [61].

## Advantages, limitations, and biosafety considerations

**Figure 5:**
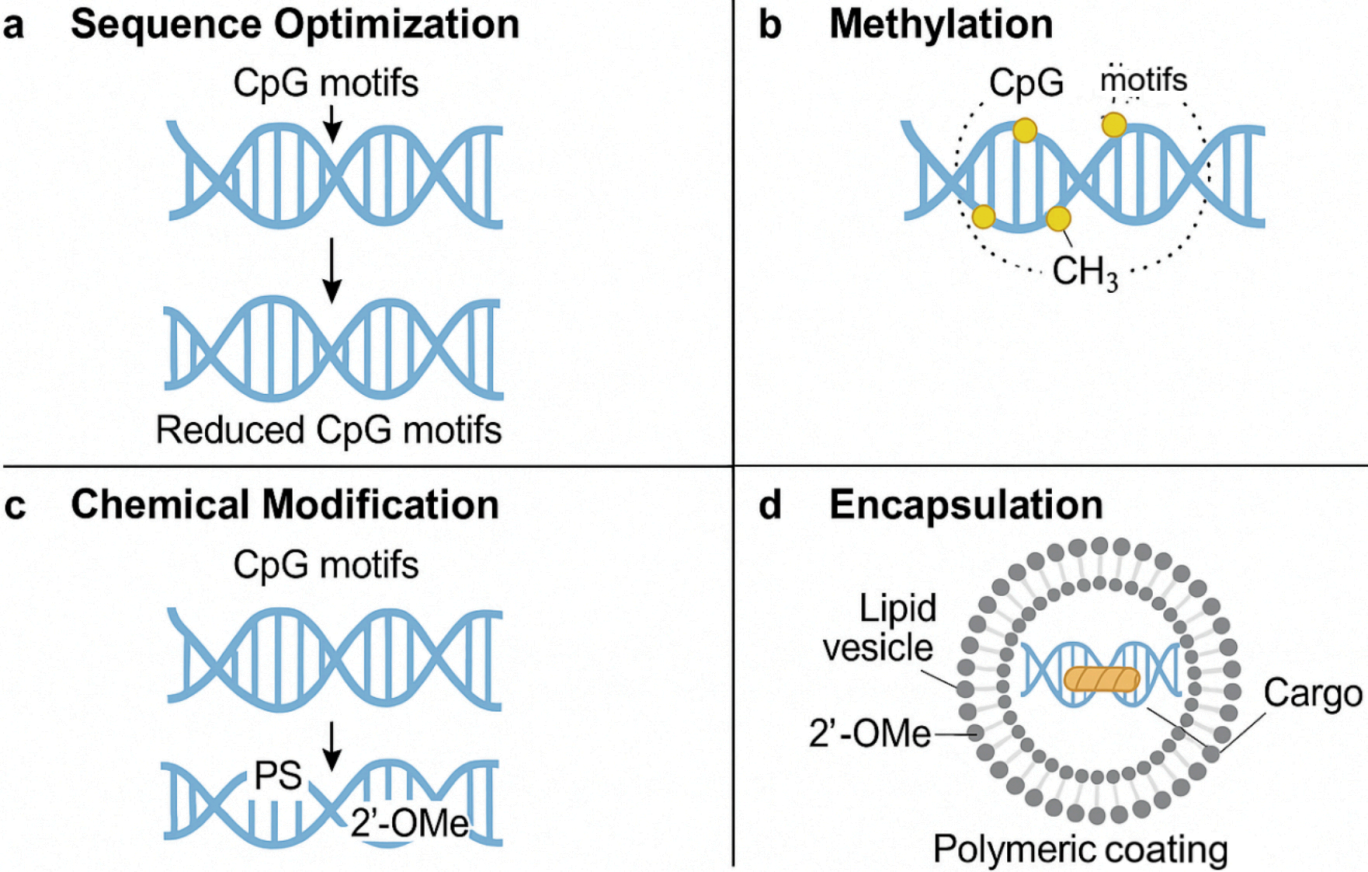
**Engineering strategies to mitigate CpG-mediated TLR9 immune activation in DNA origami nanostructures.** Approaches include (**a**) sequence optimization, (**b**) CpG methylation to mask immune recognition, (**c**) chemical modifications such as 2′-*O*-methyl or phosphorothioate substitutions, and (**d)** shielding CpG motifs with protective coatings (lipid or polymer encapsulation) to prevent direct TLR9 engagement.

The integration of DNA origami with nanofluidic systems offers a suite of advantages that surpass traditional delivery platforms in terms of structural programmability, molecular precision, and stimuli-responsive adaptability [62]. The ability to encode complex 3D geometries and functional motifs into DNA nanostructures allows for the construction of customizable carriers that can selectively interact with cellular targets, respond to internal or external stimuli (e.g., pH, temperature, enzymes, light), and release cargo with spatiotemporal accuracy [63]. These systems demonstrate enhanced biocompatibility due to the inherent biodegradability and low immunogenicity of DNA, and their negative surface charge further reduces nonspecific protein adsorption, facilitating prolonged circulation times and improved pharmacokinetics [64]. The confined environments of nanofluidic channels also enhance reaction kinetics and signal amplification, thereby enabling single-molecule sensitivity in diagnostic applications [65]. However, several limitations hinder clinical translation [64]. First, the scalability of DNA origami synthesis remains a bottleneck; current methods rely on M13-based scaffolds and expensive staple strand synthesis, limiting cost-effectiveness for large-scale production [65]. Furthermore, structural instability in physiological fluids, particularly serum nucleases and divalent ion depletion, can lead to premature degradation unless chemically modified (e.g., phosphorothioate backbones, PEGylation) or encapsulated within protective shells [66]. There are also unresolved concerns regarding endosomal escape efficiency, as only a fraction of internalized nanostructures reach the cytosol without lysosomal degradation. Immunogenicity [67]. An important immunological concern is that CpG-rich DNA origami scaffolds can trigger TLR9-mediated innate immune activation, leading to cytokine release and unwanted systemic inflammation. Several engineering solutions have been proposed to minimize this response [68]. One strategy is to employ sequence optimization to reduce the frequency of unmethylated CpG motifs without compromising structural stability. Alternatively, site-specific methylation of CpG dinucleotides has been shown to attenuate TLR9 recognition while preserving correct folding of DNA origami structures. Chemical backbone modifications, such as incorporation of 2′-*O*-methyl or phosphorothioate-modified nucleotides at CpG-rich regions, further enhance nuclease resistance and reduce immunogenicity [69]. Encapsulation within lipid vesicles or polymeric coatings can also shield CpG motifs from direct immune recognition, thereby lowering the risk of immune activation. Collectively, these approaches provide a versatile engineering toolbox for mitigating CpG-driven immunostimulation and advancing the clinical translation of DNA origami nanostructures [69]. To address concerns of CpG-driven immune activation, multiple engineering solutions have been developed, including methylation, chemical modification, and shielding with biocompatible coatings (Figure 5) [70]. Regulatory challenges also persist due to the hybrid nature of these systems, which straddle the interface of biologics, nanomaterials, and devices—raising complex questions about classification, approval pathways, and quality control standards [71]. Despite these hurdles, advances in in vivo stability engineering, AI-guided sequence design, and automated origami production (e.g., robotic DNA printers and enzymatic synthesis) are rapidly addressing current limitations, making the path toward clinical-grade DNA nanofluidic systems increasingly viable [68]. Continued refinement of biosafety profiles through long-term immunotoxicity studies and optimization of degradation kinetics will be pivotal to transitioning from experimental to therapeutic deployment [72]. Recent developments in “automatic DNA printers” and enzymatic assembly platforms have been highlighted as a potential solution for scalable origami production. However, current technologies still face important limitations that must be addressed before clinical translation is feasible. A key challenge is the synthetic error rate of oligonucleotide production, which can exceed 1%, leading to misfolding, incomplete structures, or loss of functional motifs [73]. At the gram scale, such error frequencies accumulate significantly, necessitating purification steps that increase cost and reduce throughput [74]. In addition, automated printers are currently restricted in scaffold length and staple diversity, limiting the complexity of achievable nanostructures. Error-correction algorithms, improved DNA synthesis chemistries, and enzymatic ligation strategies are under development to lower error rates and enable longer, more accurate scaffolds [74]. Coupling automated printing with high-throughput quality control systems, such as capillary electrophoresis and next-generation sequencing, will be essential to ensure reproducibility and structural fidelity at scale. While automation represents a promising direction, these technological constraints highlight the need for continued innovation in synthesis chemistry, process optimization [75], and cost reduction before DNA origami printers can support routine clinical-grade manufacturing. Table 5 provides a side-by-side comparison of DNA origami nanocarriers and conventional drug delivery systems in terms of precision, responsiveness, biocompatibility, and clinical readiness.

**Table 5:** Advantages and limitations of DNA origami-nanofluidic platforms versus traditional nanocarriers.

**Feature**	**DNA origami-nanofluidic systems**	**Traditional nanocarriers (liposomes, polymers, dendrimers, etc.)**	**Commentary**
Structural precision	Nanometer-scale programmable architecture (1–2 nm resolution)	Limited geometric control; dependent on self-assembly or bulk properties	DNA origami offers atomic-level customizability not achievable in conventional carriers
Cargo loading specificity	Precisely addressable sites; multiplexed cargo placement	Nonspecific encapsulation; batch-to-batch variability	Origami allows for stoichiometric and directional loading
Stimuli responsiveness	Highly tunable: pH, redox, enzyme, light, thermal, electric field	Limited to pH, enzyme, and sometimes redox	Origami systems integrate complex logic-based responsiveness
Biocompatibility and degradability	High; composed of natural DNA; easily metabolized	Variable; dependent on material (e.g., PEG, PLGA, chitosan)	DNA origami generally elicits lower inflammatory responses
Intracellular targeting	Site-specific via aptamers, DNAzymes, logic gates	Targeting via antibodies, peptides, or the passive EPR effect	Origami allows AND/OR gate logic for precise cell type recognition
Fabrication scalability	Limited; costly oligonucleotide synthesis and thermal folding	High; scalable emulsification and self-assembly	A key limitation for origami platforms in clinical translation
Stability in physiological conditions	Susceptible to nuclease degradation without modifications	Generally stable depending on lipid/polymer coating	Chemical modifications (e.g., PEGylation) improve origami performance
Endosomal escape efficiency	Moderate; dependent on auxiliary strategies (e.g., fusogenic peptides)	Often enhanced via pH-sensitive polymers or ionizable lipids	Both require additional design elements for efficient cytosolic delivery
Immunogenicity	Low to moderate (sequence- and CpG-dependent)	Variable; can induce cytokine release or complement activation	CpG content and repetitive motifs in DNA origami require optimization
Clinical translation readiness	Emerging; preclinical proof-of-concept demonstrated	Advanced; several platforms FDA-approved (e.g., liposomes)	Origami-nanofluidics are still in early translational stages

EPR: enhanced permeability and retention; PEG: polyethylene glycol.

## Metabolic kinetics and pharmacokinetic considerations

Metabolic kinetics and systemic pharmacokinetics are pivotal determinants of the clinical translation of DNA origami nanostructures, yet they remain relatively underexplored [76]. Available studies suggest that biodistribution is strongly dictated by geometry, size, surface charge, and chemical modification of the constructs. Small origami structures below the renal filtration threshold (typically < 6–8 nm hydrodynamic diameter) undergo rapid glomerular clearance and appear in urine within hours, whereas larger architectures (> 50–100 nm) evade renal elimination but accumulate in the liver and spleen through uptake by Kupffer cells and the mononuclear phagocyte system [76]. The half-life of DNA origami nanostructures in circulation is generally short, ranging from minutes to a few hours, primarily due to nuclease-mediated degradation and recognition by serum proteins [77]. For instance, tetrahedral origami frameworks have been reported to exhibit blood half-lives of 1–2 h in mice with significant accumulation in kidney and liver tissues, while rod-like or tubular geometries show preferential hepatic uptake [78].

Surface engineering significantly alters these pharmacokinetic outcomes. PEG modification reduces protein adsorption and prolongs systemic circulation, while protective coatings such as lipids or polymers mitigate nuclease degradation and modulate biodistribution [79]. Ligand conjugation (aptamers, antibodies, peptides) enhances receptor-mediated endocytosis in target tissues but may accelerate hepatic clearance depending on the receptor density and expression. In terms of metabolism, circulating origami structures are gradually degraded by endonucleases and exonucleases into oligonucleotide fragments, which are subsequently excreted via renal or biliary pathways [80]. Despite encouraging early results, systematic pharmacokinetic studies of DNA origami remain sparse compared to traditional nanocarriers. Key parameters such as volume of distribution, clearance rate constants, and tissue-specific accumulation have not yet been comprehensively profiled across different geometries and functionalizations [81]. Quantitative data on long-term clearance, repeated dosing, and chronic accumulation are also limited, which presents a barrier for regulatory acceptance [82]. The application of real-time imaging and tracking techniques, including radiolabeling, fluorescence tomography, and isotope tracing, could provide much-needed insights into absorption, distribution, metabolism, and excretion profiles. Such studies will be critical for establishing predictive pharmacokinetic models and designing clinically viable origami-based nanomedicines [82].

## Author’s outlook

The convergence of DNA origami nanotechnology with nanofluidic engineering represents a paradigm shift in how we conceptualize and implement targeted intracellular delivery. Unlike conventional nanocarriers, which rely on passive diffusion or generalized targeting, DNA origami-based systems offer programmable precision at the atomic scale—capable of executing complex, stimulus-driven logic within the cellular environment. When integrated into smart nanofluidic platforms, these systems gain fluidic controllability, enabling real-time modulation, guided navigation, and multi-stage release mechanisms that respond to biochemical cues such as pH, enzymatic activity, redox potential, and even external fields like NIR light or electric pulses. Such capabilities not only improve therapeutic specificity and efficacy but also reduce systemic toxicity, addressing longstanding limitations in oncology, gene therapy, and immunotherapy.

Looking ahead, we foresee the evolution of these platforms into autonomous, self-regulating nanorobotic systems capable of real-time decision-making in vivo. Advances in synthetic DNA chemistry, AI-guided origami sequence design, and microfluidic circuit integration will enable next-generation therapeutic machines—engineered to sense, compute, and act within the molecular context of diseased cells. Furthermore, the integration of DNA origami nanofluidic devices with wearable biosensors and digital health infrastructure could lead to closed-loop feedback systems, where diagnostics and therapeutics are dynamically co-regulated. While challenges such as large-scale production, long-term biosafety, and regulatory classification remain, rapid technological maturation suggests that clinical translation is not only feasible but imminent. In essence, smart nanofluidic DNA origami systems are more than carriers—they are the foundation of an emerging era of programmable medicine, where disease treatment is adaptive, personalized, and molecularly intelligent.

## Conclusions

Smart nanofluidic systems incorporating DNA origami nanostructures represent a remarkable leap forward in the field of targeted intracellular delivery. These platforms bridge the gap between synthetic nanosystems and biological functionality, leveraging the molecular precision of DNA origami with the fluidic control and environmental adaptability of nanofluidic channels. Throughout this review, we have highlighted how programmable DNA assemblies can be rationally designed to respond to endogenous and exogenous stimuli—such as pH, enzymatic activity, redox potential, or light—thereby enabling cargo release with exceptional spatial and temporal resolution. When embedded into nanofluidic architectures, these origami constructs can be directed, actuated, and modulated through electrophoretic or thermal cues, making them uniquely suited for dynamic biological environments.

Preclinical studies have underscored their utility in oncological applications, gene editing, and immunomodulation, showing superior specificity and therapeutic efficacy compared to traditional nanocarriers. However, several translational barriers persist, including nuclease sensitivity, immunogenicity, and manufacturing scalability. The field is rapidly advancing to address these issues through chemical modifications, encapsulation techniques, and scalable synthesis using automated origami printers. Additionally, future integration of artificial intelligence, microelectromechanical systems (MEMS), and bioinformatics-driven sequence design holds promise for real-time adaptable therapeutics tailored to patient-specific molecular profiles.

Ultimately, the integration of DNA origami into smart nanofluidic systems offers a new paradigm in personalized medicine—one where intracellular delivery is not only targeted but autonomous, responsive, and programmable. As research progresses, these hybrid systems are poised to revolutionize the way we approach precision therapy, diagnostics, and molecular-level intervention in complex diseases.

## Abbreviations

3D: three-dimensional
BBB: blood-brain barrier
GSH: glutathione
PEG: polyethylene glycol

## Declarations

### Copyright

© The Author(s) 2025.
